# Mid-pregnancy ewe shearing and the effects on fetus liver and muscle glycoprotein deposits

**DOI:** 10.21451/1984-3143-AR2018-0084

**Published:** 2018-12-05

**Authors:** João Carlos Morini, Adriana Caroprezo Morini, Lina Castelo Branco Motta, Carlos Eduardo Ambrosio, Phelipe Oliveira Favaron, Flávia Thomaz Verechia Rodrigues, Luiz Alberto Oliveira Ribeiro, Luciana Relly Bertolini, Marcelo Bertolini, Maria Angélica Miglino, Pedro Primo Bombonato

**Affiliations:** 1 Faculty of Veterinary Medicine and Animal Science, São Paulo University, São Paulo, Brazil.; 2 Federal University of West of Pará, Santarem, Pará, Brazil.; 3 Faculty of Animal Science and food Engineering, São Paulo University, Pirassununga, São Paulo, Brazil.; 4 Paulista State University, Dracena, São Paulo, Brazil.; 5 Federal University of Rio Grande do Sul, Rio Grande do Sul, Brazil.; 6 University of Fortaleza, Ceará, Brazil.

**Keywords:** bone, glycoprotein, lamb, muscle, placenta

## Abstract

The reason why shearing ewes in mid-pregnancy does increase the lamb birth weight is not completely clears. Therefore, we focused on the analyses of the deposition of glycogen in different fetal tissues to investigate this issue. Thirteen pregnant Australian Merino ewes, raised in native pasture, were separated in two groups. One group (n = 7) was shorn (SE) at 70 days of pregnancy, whereas another group (n = 6) remained unshorn (NSE). Cesarean section was conducted in all the ewes at near parturition, when placenta and fetuses sampling were collected. Placenta, liver and muscle samples were fixed and stained with glycoprotein-reactive acid-Schiff acid for analysis under light microscopy. The quantification of these glycoproteins was performed with the support of a program that analyzes the measurement of the intensity of staining by field. Five random fields from each sample were used, where statistical analyzes was used as normal test T. Among the analyzed regions, the deposition of glycoprotein between SE and NSE groups was statistically different in the hepatic portal vein (54,499.23 µm^ 2^ in SE and 34,830.73 µm^2^ in NSE) and in the total muscle area of the sample fragment (41,128, 7 µm^2^ and 31,942.7 µm^2^ , respectively; P < 0.05). We conclude that shearing ewes at the 70th day of gestation lead to accumulation of glycoproteins in the liver and muscle of fetuses, which may be responsible for the increase in birth weights in that group.

## Introduction

The sheep farming has high social and economic importance, since it is an agricultural activity that originates various products such as meat, dairy products, wool and leather. However, a problem faced by this industry is reproductive losses, represented by high mortality rates of newborns (Dwyer *et al*., 2006).

Adverse climatic conditions and low birth weight are the main causes of death of lambs in the perinatal period ( Labeur *et al*., 2017 ; Godfrey *et al*., 2017 ; Elizalde *et al*., 2018 ). In cold regions, the condition tends to worsen, since low body weight may cause hypothermia, since lambs after birth do not have enough surface area to perform heat exchange, besides having low energy reserves to control their temperature ( McCoard *et al*., 2014 ). It was verified that the shearing of sheep during pregnancy causes the birth of lambs of greater body weight ( Morris and Mccutcheon, 1997 ). Lauber *et al*. (2017) have found that lambs born to ewes shorn in late-pregnancy are better able to maintain body surface temperature than those derived from unshorn ewes. However, the cause for such an event is not completely clear. Decades ago, some authors have associated such event to placental factors. Since placental growth is completed around the 90th day of pregnancy, shearing on day 70 could change the maternal metabolism, leading to an increase in glucose supply to the uterus and, consequently, to the fetus ( Alexander, 1964 ; Symonds *et al*., 1986 ). Such change could be responsible for an increase in placental size enhance and fetal weight.

Based on that, this work aimed to evaluate whether shearing pregnant ewes would have any effect on fetal liver and muscle glycoprotein deposits (GAD).

## Material and methods

All procedures were approved by the Bioethics Commission of the Faculty of Veterinary Medicine and Animal Science of the São Paulo University, nº 1157/2007.

A flock of 21 Australian Merino ewes were raised under native pasture of the Brazilian Lutheran University, located in the state of Rio Grande do Sul, Brazil. Mating was carried out by two ram, the day of mating being considered as day zero. After 15 weeks, ultrasound examinations were performed, confirming positive pregnancy in 13 ewes, all with single fetus. These ewes were divided into two groups: the first (n = 7) was shorn (SE) with 70 days gestation, while the second (n = 6) remain unshorn (NSE). Near the period of parturition, around 140 days of gestation in relation to the day of each mating, the cesarean sections were performed in all the ewes, where the placenta and the fetuses were collected.

Fetuses and placentas were weighed, and fragments of the fetal left *Rectus femoris* muscle, liver and samples of placentomes (both the maternal - caruncle

- as well as the fetal - cotyledon) were collected. Samples were cut and reduced tot the size of 1 cm. Then, fragments were fixed in Bouin alcohol, with either 4%parafomoldehyde or 10% formaldehyde, both made in sodium phosphate buffer (PBS).

After fixation, samples were washed in PBS, dehydrated in an ethanol series of increasing concentration (70 to 100%), to be diaphanizated in xylol and included in paraplast. Sections of 5 μm were made using a microtome (Leica – RM2065, Berlim Germany). To analyze a larger area of the fragments at different locations in each block cut out material follows five sequential sections were produced subsequently kept the blades cut from 100 microns to produce more and we returned five sequential slides this method is repeated in each block 3 times

The slides for morphological description for placenta tissue were stained with hematoxylin-eosin (HE) and analyzed under light microscopy. The technique of periodic acid-Schiff (PAS) for histochemistry reaction was used to visualize the accumulation of glycoproteins.

**Figure 1:**
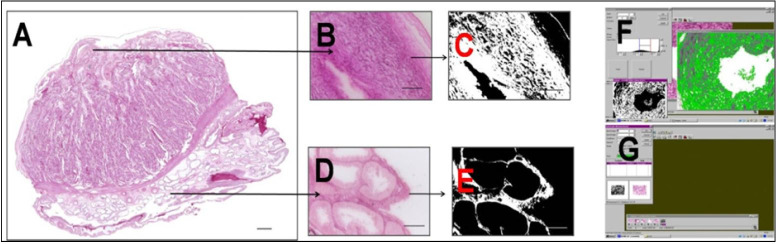
Used methodology for glycogen deposit evaluation. A: Ovine placentome, PAS, 200 µm; B and D are examples of random measured areas that were analyzed, PAS, 25 µm; C and E were quantified areas in white after phase contrast image; 25 µm; F and G are examples of the specific program for area measurement.

To assess the amount of glycoprotein in the slides stained with PAS, five fields of 50 μm ^2^ per animal were measured, with 40x of magnification. These fields were uniform and from different histological sections of each sample. The image of each field was obtained through an Axioscopie Zeiss® optical microscope and analyzed in a microcomputer with a specific program for morphometry (Kontron KS-400 3.0), which allows the measurement of the glycoprotein through the difference in color intensity in the different regions of the field analyzed. The color intensity was measured from the intensity of the reaction of PAS with glycoprotein, therefore, areas with greater amount of glycoproteins were marked more strongly. Selecting the region stained with PAS in each of the fields it is possible to obtain an average value of color intensity, consequently, the quantity of glycogen present in that area. ( Fig. 1 ).

In the placentomas, the glycoproteins were analyzed in the villi and endometrial glands, besides the vessels, epithelium and mesenchyme, both maternal and fetal. In the liver tissue, the analyzed areas were the portal triad, portal vein, center lobular vein, sinusoids and hepatocytes, whereas on the muscle tissue, fibers, vessels and fat tissue were evaluated. In the rectus femoris muscle, the analysis of the disposition of the muscular fibers between the groups was performed, besides the deposition of glycoproteins in fragments of the muscular vessels and the adipose cells of the muscles.

The results were presented descriptively with mean ± standard deviation (SD). The normality of the data was verified by the Kolmogorov-Smirnov test, adopting for the analysis of the means of the groups the test T, being performed with the RGui version 2.6 program (R-project, University of Auckland, New Zealand), with the significance level of 5%.

## Results

The mean weights of the fetuses were 3,688 kg in the SE group and 3,596 kg in the NSE group, while the average placental weights were 2,287 kg and 1,923 kg, respectively. Despite the difference in body and placental weights between the groups, these values were not statistically significant (P > 0.05).

**Figure 2:**
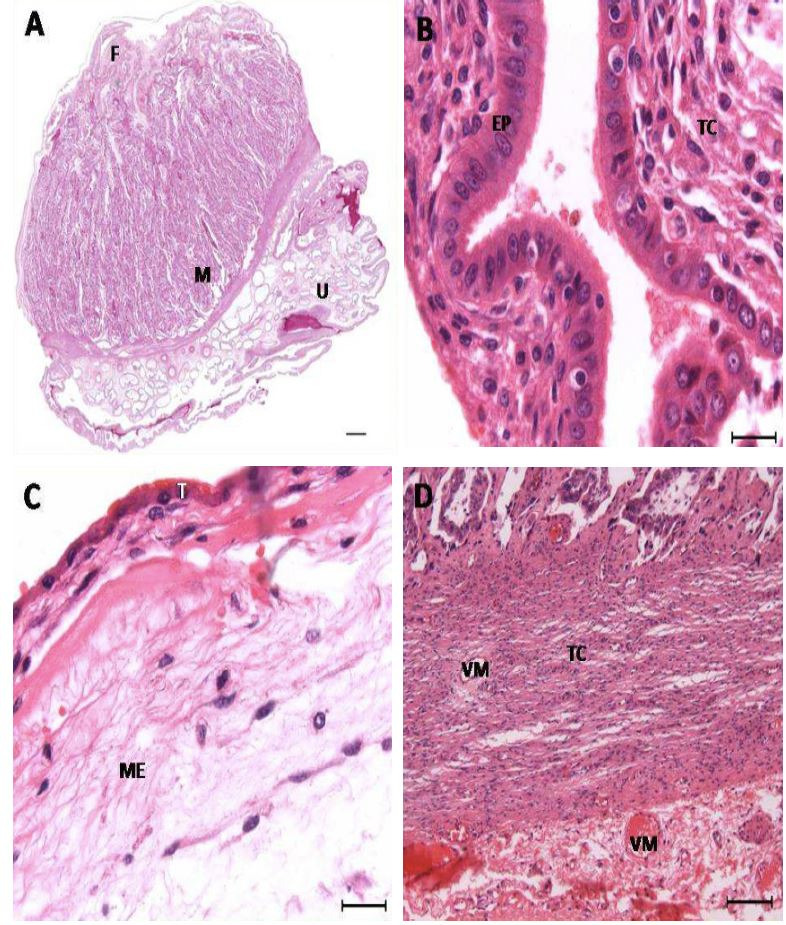
Photomicrographs of the areas studied in ovine placentones. A: panoramic view of the placentome of sheep stained by PAS; B: maternal epithelium stained by HE; C: fetal epithelium stained by HE; D: maternal connective tissue. F: fetal margin; M: maternal margin; U: uterine margin; EP: uterine epithelium; TC: connective tissue; T: trophoblast; ME: mesenchyme; VM: maternal vessel. Bar A: 500 μm; B and C: 20 μm; D: 100 μm.

**Figure 3:**
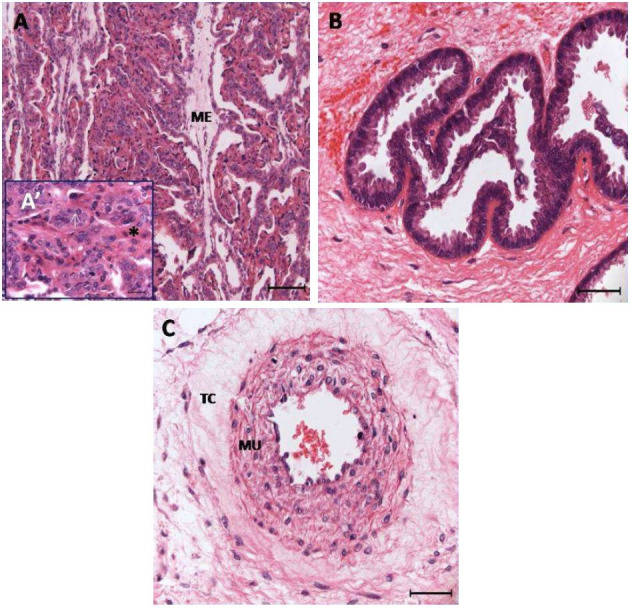
Photomicrographs of the areas studied in sheep placentones. A: placentomial villi; A': maternal and fetal part of villus; B: endometrial glands; G: fetal muscular artery. ME: mesenchyme; arrow-fetal villus; * maternal villus; TC: connective tissue; MU: muscular. Stained with HE. Bar A: 100 μm; A': 20 μm; B and C: 40 μm.

The placentones, both in the maternal and fetal regions, presented a simple cubic epithelium ( Fig. 2 ), rich in vessels in the connective tissue ( Fig. 3C ) with villi on both sides ( Fig. 3A ). The placental endometrial glands were provided with simple cubic epithelium surrounded by connective tissue ( Fig. 3B ). All other analyzed fields of the placenta had higher deposition of glycoprotein in sheared group, but without statistical differences between the groups, as shown in Table 1 .

**Table 1:** Average of the GAT area (µm^2^) found in placenta between the groups shorn at 70^th^ day of gestation (SE) and unshorn pregnant ewe (NSE).

		SE	NSE
Vessel	(M)	36131 ± 4416 ^a^	38740 ± 8075,4^a^
	(F)	46076 ± 17972,3 ^a^	45304 ± 9034,9^a^
Epithelium	(M)	28067 ± 10929,5 ^a^	24841 ± 5971,5^a^
	(F)	35634 ± 18102 ^a^	23435 ± 262,1^a^
Mesenchyme	(M)	43459 ± 16849,9 ^a^	33675 ± 6583,7^a^
	(F)	40863 ± 18729,2 ^a^	30412 ± 7862,8^a^
Vilosities		41682 ± 13210,2 ^a^	35636 ± 12521,1^a^
Endometrial glands		21496 ± 9037,8 ^a^	20704 ± 8794,3^a^

* P ≤ 0.05; averages with distinct letters on lines are different by significance by T student statistical test. M: maternal part; F: fetal part.

**Figure 4:**
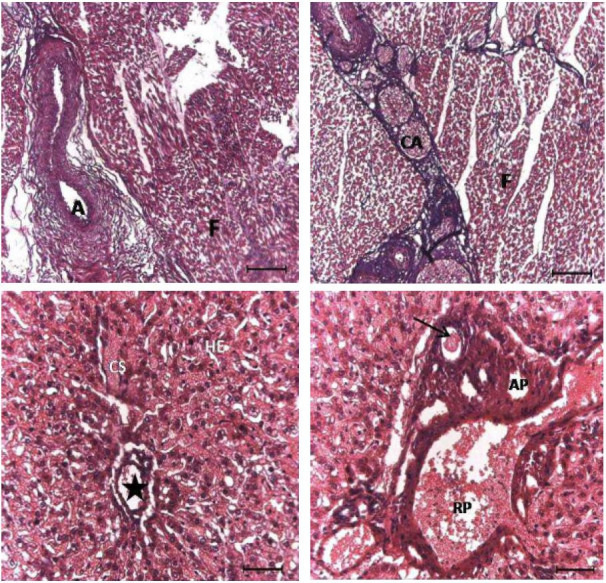
Photomicrographs of the areas studied in the ovine muscle (A and B) and liver (C and D). A: elastic artery; B: adipocyte cells; C: lobular center vein, sinusoid capillary; D: portal triad. A (black): elastic artery; CA: adipocyte cells; CS: sinusoidal capillary; F: muscle fiber; HE: hepatocytes; star: lobular center vein; arrow: bile duct; AP: hepatic artery; VP: portal vein. Stained by HE. Bar A and B: 100 μm; C and D: 50 μm.

The livers of the fetuses derived from both groups had characteristic structural composition, with central lobular veins, sinusoidal capillaries, and hepatocytes in the parenchyma ( Fig. 4C and 4D ). In the portal triad, more specifically in the hepatic portal vein, glycoprotein quantified in the SE group was significantly higher than in the NSE group ( Tab. 2 ). Like the portal vein, another site where a significant statistical difference between the groups was noted was the *Rectus femoris* muscle in its total area, where the SE group had a higher deposition of glycoprotein in comparison to the fetal muscle of the group NSE, as shown in Table 2 . The muscle fibers and adipocytes found in both groups had similar composition ( Fig. 4A and 4B ).

**Table 2:** Results (Average, Max and Min) of GAT in liver portal vein area and *Rectus femoris* muscle area (µm^2^) of fetus from pregnant ewe shorn at 70^ th^ day of gestation (SE) and unshorn pregnant ewe (NSE).

	Liver portal vein		Fetal *Rectus femoris* muscle	
	SE	NSE	SE	NSE
Average	54499 ± 3020,9^a^	34831 ± 6470,5^b^	41129 ± 1715,7^a^	31943 ± 4054^b^
Max	58419	39526	42737	37509
Min	51026	25681	38751	27970

* P ≤ 0.05; averages with distinct letters on lines are different by significance by T student statistical test.

## Discussion

Thermal stress is a common problem in sheep farming ( Godfrey *et al*., 2017 ). Survival capacity is intertwined with the lamb's response to the environment in which it is born (Dwyer *et al*, 2006). It is often found in cold climatic conditions, where the lamb has a low percentage of body fat in relation to its surface area, which increases heat loss ( McCoard *et al*., 2014 ), also causing differences in metabolic rates, consequently in the available energy reserves (caused by the tremors) ( Labeur *et al*., 2017 ). The shearing of sheep during gestation has been commonly used as an alternative because it is capable of altering the placental metabolism (Black *et al*., 1990), consequently the body weight of lambs ( Ribeiro *et al*., 2010 ). However, the motive responsible for this fact is not fully understood.

When analyzing the levels of glycoproteins, we found that they were in a higher concentration in the SE group, thus suggesting that they may be responsible for the increase in body weight. The concentration range of glycoproteins analyzed in our study indicates that the glycoproteins accumulate in the fetus during gestation and may be directly related to the stress caused by shearing during the gestational period, because Roussel *et al*. (2004) verified the increase of cortisol in lambs with higher body weight. Barbieri *et al*. (2018) found that there is an increase in placental weight and the number of cotyledons in sheared sheep in the middle of pregnancy, which may increase levels of nutrient transfer between the sheep and the fetus. Supporting the hypothesis of this author, in the present study, the placental weight of the sheared group was higher, that is, the fetal weight may be being increased by the increase of nutrient transfers, consequently, of glycoproteins. Guyoti *et al*. (2015) also verified that sheep shearing affected the hematological parameters of the lambs, hematocrine and hemoglobin values being lower in shear groups. This author cited that the greater amounts of hematocrine in lambs derived from non-sheared sheep may have occurred as a compensatory effect of decreased blood distribution to the fetus, consequently the resulting hypoxia may lead to less placental growth.

Among the structures studied in the liver, the portal vein showed significant differences between SE and NSE groups. That vessel carries 80% of the blood to the liver, consisting of little amount of oxygen, but rich in nutrients ( Junqueira and Carneiro 2004 ). The nutrients may be responsible for the significant difference in the amounts of glycoprotein between groups. Since during gut absorption the liver captures and converts glucose into glycoprotein and triglycerides ( Cunningham, 1999 ) and as the shearing protocol results in an increase in fetal weight (Morris and McCuttheon, 1997; Banchero *et al*., 2010 ; Guyoti *et al*., 2015 ; Barbieri *et al*., 2018 ), it is highly possible that shearing in our study was associated with higher concentration of glycoprotein in the liver of fetuses from shorn ewes.

In the fetal *Rectus femoris* muscles in the SE group, large amounts of glycoprotein were found, being significantly different from the control group. This significance may be related to the development of the fetus. According to Toboga *et al*. (2003) , the glycoprotein is metabolized quickly as needed when it is required, and when the fetus is larger, it needs a higher demand of glycoprotein.

Insulin is the ideal substance because it is the physiological regulators, exerting a number of simultaneous effects on interconnected metabolic pathways (Wiernsperger, 2005). The reduced insulin secretion may have reduced glucose utilization by maternal tissues, thus sparing glucose for use by the placental/fetal unit ( Revell *et al*., 2000 ). In Wiernsperger (2005), it was suggested that the reduced insulin response to glucose challenge, alternatively, may have been due to an increased sensitivity of maternal tissues to insulin such that less quantities was required to clear the glucose load. Consequently, the insulin challenges caused the same reductions in plasma glucose concentrations in all ewes studied, which did not support the concept of altered insulin sensitivity. The non-insulin dependent uptake of glucose across the placenta is an important factor described in the literature as a likely candidate to account for a more rapid glucose clearance from the maternal circulation ( Revell *et al*., 2000 ). The author suggested that the facilitated diffusion of glucose across the placenta was enhanced by shearing in mid-pregnancy, leading to an increase in the maternal-fetal gradient in glucose concentration, which would normally be associated with an increase in maternal glucose concentration.

A number of studies have showed that the mid-pregnancy shearing resulted in increase in the birth weight of both singleton and twin lambs. Corner *et al*. (2007) showed that increases in lamb birth weight have generally been reported in either singleton ( Morris *et al*., 2000 ; Kenyon *et al*., 2002 ; Revell *et al*., 2002 ) or twin-born lambs ( Morris and McCutcheon, 1997 ; Smeaton *et al*., 2000 ; Kenyon *et al*., 2004 ), but less frequently for both ( Kenyon *et al*., 2002 ; Corner *et al*., 2006 ). According to results obtained by Revell *et al*. (2000) , the shearing protocol results in an increase of over 1.0 kg in the mean birth weight of twin lambs in response to mid-pregnancy shearing, without any change in the birth weight of singletons. Revell *et al*. (2002) showed that the increase in lamb birth weight was due, in part, to an increase in gestation length of 1.2 days. Shearing ewes during pregnancy resulted in an increased gestation length of approximately 1 day. These results indicate that mid-pregnancy shearing can increase lamb birth weight without increasing ewe herbage intake or placental weight. An increase in the efficiency of nutrient uptake by the placenta is implied, and possible effects on the activity of thermogenic tissues are discussed. The placental weight as well as the study of Revell *et al*. (2002) was also higher in the clipped traces showing that glycoproteins and nutrient uptake and transported through the placenta to the fetus may be responsible for the weight gain of the fetus at birth.

Alternatively, shearing during mid-pregnancy has also been shown not to alter the proportion of lambs born weighing less than 3.5 kg ( Corner *et al*., 2007 ).

The author suggested that mid-pregnancy shearing did not alter the fetal growth rate of small lambs. Some similar information was described by Guyot *et al*. (2015). However, Kenyon *et al*. (2006) reported mid-pregnancy shearing to result in an increase in the survival rate of twin and triplet lambs due to a reduction in the proportion of lambs that weighed less than 3.5 kg at birth, which is economically important to the sheep industry. Despite the small difference of 100 grams in weight of the media made between the groups in our work, we work with lambs at 6 pounds, close to the results presented in the work of Corner *et al*. (2007) , although none of our animals have reached the final stage of pregnancy and rising off the weight was quite close to the findings of this author, both in late pregnancy in which he found in the middle of gestation was 3.5 kg and next to our work. Thus agreeing with the findings of the Kenyon *et al*. (2006) shearing performed during pregnancy causes increase in birth weight of lambs. We cannot say that the differences found in glycoprotein deposition levels are the only ones responsible for the increase in fetal weight, but we can say that there is an accumulation of these in body fat, thus making the lamb heavier.

In conclusion, after studying the deposits of glycoproteins in placental and fetal tissues of shorn and unshorn Australian Merino ewes on day 70 of gestation, but the concentration of glycoproteins it is increased in sheared groups. The largest concentration of glycoprotein in the liver fetal occured around the fetal blood vessels, more specifically in the pericyte branches of the hepatic portal vein. Lambs born from ewes shorn on day 70th of pregnancy showed a higher deposition of muscle glycoprotein. The difference in concentration of fetal liver glycoprotein in the muscle and femoral tissue of lambs, confirms the hypothesis that shearing ewes on day 70 of pregnancy increases birth weight.

References
